# Tropical instability wave modulation of chlorophyll-a in the Equatorial Pacific

**DOI:** 10.1038/s41598-021-01880-5

**Published:** 2021-11-18

**Authors:** Wei Shi, Menghua Wang

**Affiliations:** 1 NOAA National Environmental Satellite, Data, and Information Service, Center for Satellite Applications and Research, E/RA3, 5830 University Research Ct., College Park, MD 20740 USA; 2 CIRA at Colorado State University, Fort Collins, CO USA

**Keywords:** Marine biology, Physical oceanography

## Abstract

The global daily gap-free chlorophyll-a (Chl-a) data derived using the data interpolating empirical orthogonal functions (DINEOF) technique from observations of the Visible Infrared Imaging Radiometer Suite (VIIRS) in 2020 and the in situ measurements at the Tropical Ocean Atmosphere (TAO) moorings are used to characterize and quantify the biological variability modulated by the tropical instability wave (TIW). Our study aims to understand how ocean physical processes are linked to biological variability. In this study, we use the TAO in situ measurements and the coincident VIIRS Chl-a data to identify the mechanism that drives ocean biological variability corresponding to the TIW. Satellite observations show that the TIW-driven Chl-a variability stretched from 90°W to 160°E in the region. The enhanced Chl-a pattern propagated westward and moderately matched the cooler sea surface temperature (SST) patterns in the Equatorial Pacific Ocean. In fact, the Chl-a variation driven by the TIW is about ± 30% of mean Chl-a values. Furthermore, the time series of Chl-a at 140°W along the equator was found to be in phase with sea surface salinity (SSS) at 140°W along the equator at the TAO mooring since late May 2020. The cross-correlation coefficients with the maximum magnitude between Chl-a and SST, Chl-a and SSS, and Chl-a and dynamic height were –0.46, + 0.74, and –0.58, respectively, with the corresponding time lags of about 7 days, 1 day, and 8 days, respectively. The different spatial patterns of the cooler SST and enhanced Chl-a are attributed to the phase difference in Chl-a and SST. Indeed, a Chl-a peak normally coincided with a SSS peak and vice versa. This could be attributed to the consistency in the change in nutrient concentration with respect to the change of SSS. The vertical distributions of the temperature and salinity at 140°W along the equator reveal that the TIW leads to changes in both salinity and nutrient concentrations in the sea surface, and consequently drives the Chl-a variability from late May until the end of the year 2020.

## Introduction

The tropical instability wave (TIW) is a westward propagating long wave pattern of sea surface temperature (SST) in the eastern Equatorial Pacific Ocean during the period between May and December each year^1,2^. The TIW extends about 1000–2000 km with two wave modes in the period of 17 and 33 days^3^. The cusp-shaped distorted SST can have a variability of 3°C^4^. The phase speed of the TIW can range from about 0.3 m/s to 1.0 m/s in different latitudes from various observations^3–5^. TIWs are typically most intense between 160° and 100°W from July through the end of the year^6^.

The TIW activity is closely related to the El Niño–Southern Oscillation (ENSO) intensity with higher activity in the La Niña years and lower activity in El Niño years^6,7^. The strong TIWs can cause significant atmospheric response in the tropical Pacific^8^. On the other hand, the properties of TIWs are further influenced by the modulation of the TIW-driven surface wind-stress^9^. Recent model studies show that the TIW in the Equatorial Pacific Ocean is generated from an unstable mode resulting from the coupling of two Rossby waves^10^. The TIW forms as resonance develops between two equatorial Rossby waves when the background currents slowly increase^11^. The TIW induces the maximum subsurface velocity oscillation at a depth of 70–90 m with a magnitude of 0.1–0.2 m/s^12^. The TIW temperature and velocity variabilities with periods of 17 and 33 days were observed in the subsurface at 140°W^13^. Furthermore, the tropical instability vortices (TIVs) initiated from the TIW propagate westward as a train of anticyclonic eddies^14^.

As the most effective way to study the large-scale ocean processes, satellite remote sensing has been used to study the TIW. Both the geostationary and the microwave SST data can detect and characterize the SST variability modulated by the TIW^1,2,4^. The TIWs are also related to the sea surface height (SSH) from satellite observations^15,16^. A strong dynamical influence of TIWs within 5° of the equator is revealed with the SST and SSH observations. It is also reported that sea surface salinity (SSS) variability was driven by the TIWs from the Aquarius Satélite de Aplicaciones Científicas-D (SAC‐D) satellite observations^5^. The SSS anomaly caused by the TIWs has a magnitude of about 0.5 psu, and the dominant propagation speed near the equator as revealed by the SSS measurements is about 1.0 m/s.

As a major ocean phenomenon in the Equatorial Pacific Ocean, the TIWs can not only lead to a significant variability of physical parameters such as temperature and salinity, but also modulate the biological and biogeochemical processes in the region. Indeed, enhanced chlorophyll–a (Chl-a) concentration is associated with the TIWs in the Equatorial Pacific Ocean^17,18^. On the other hand, Chl-a concentration is also critical when conducting large-scale modelling studies in the tropical Pacific^19–21^. The Chl-a variability driven by the TIW also influences both the intensity of TIW and the large‐scale SST in the tropical Pacific Ocean^22^. In fact, positive feedback on the ENSO is observed due to the TIW-induced Chl-a effect^23^.

Unlike the microwave SST, SSS, and SSH altimeter from active sensor measurements, which can usually provide observations regardless of the atmosphere condition, satellite ocean color measurements are limited to only clear sky conditions. Chl-a changes associated with the TIWs were only reported in some clear daily snapshots of the satellite ocean color observations in the Equatorial Pacific Ocean^17,18^. It is difficult to address the TIW-modulated biological variability and the driving mechanism quantitatively due to frequent cloud coverage in the Equatorial Pacific Ocean.

Recently, the daily global gap-free Chl-a data are being routinely produced by the NOAA Ocean Color Team using the data interpolating empirical orthogonal functions (DINEOF) method^24,25^ from measurements of the Visible Infrared Imaging Radiometer Suite (VIIRS) onboard the Suomi National Polar-orbiting Partnership (SNPP) and the NOAA-20 satellites. In this study, we analyze VIIRS daily gap-free Chl-a data in the year 2020 over the Equatorial Pacific Ocean to evaluate how the TIWs modulate the biological activities in the region, e.g., Chl-a magnitude change, temporal variation, spatial extent, Chl-a anomaly pattern propagating phase speed, etc. In addition, the in situ measurements of water temperature, salinity, and dynamic height at the one of the Tropical Atmosphere and Ocean (TAO) buoy arrays are examined to identify the mechanism that drives the Chl-a dynamics due to the TIW.

## Data and methods

### VIIRS-derived DINEOF Chl-a product

Since their launches in late 2011 and 2018, VIIRS SNPP and NOAA-20 have been providing a continuous operational satellite data stream to monitor the global ocean, atmosphere, land and cryosphere^26^. As the base for VIIRS ocean color products, high-quality normalized water-leaving radiance *nL*_*w*_(*λ*) spectra^27–29^ are produced with the Multi-Sensor Level-1 to Level-2 (MSL12) ocean color data processing system^30^ at NOAA after the VIIRS measurements were vicariously calibrated on-orbit with the in situ *nL*_*w*_(*λ*) measurements at the Marine Optical Buoy (MOBY)^31,32^. The ocean color index (OCI) Chl-a algorithm^33^ is used to generate Chl-a product for the global ocean and inland waters.

Since there are always many missing pixels in the original VIIRS-measured ocean color products due to issues such as clouds, high sun glint, large solar- and sensor-zenith angles, etc.^34,35^, the DINEOF method is used to fill the missing data gaps in VIIRS-derived Chl-a data (from the merged VIIRS SNPP and NOAA-20 Chl-a data)^24,25^. The DINEOF-produced gap-free daily Chl-a data have a comparable accuracy to the original data^24,25^. Therefore, VIIRS-derived gap-free daily Chl-a data can be used to continuously monitor changes in the global ocean^36^ and study short term ocean dynamics such as ocean diurnal variations^37^.

In this study, VIIRS-measured daily gap-free Chl-a data^24,25^ acquired during 2020 in the Equatorial Pacific Ocean are used to study the Chl-a variability modulated by the TIW. The region with Chl-a affected by the TIW is identified. The spatial and temporal variabilities of the TIW-driven Chl-a change are characterized and quantified. The period and the propagating speed of the cusp-shaped Chl-a peaks and troughs are estimated. In combination with SST measurements and in situ temperature and salinity profiles, the driving mechanism for the TIW-driven Chl-a variability is further addressed and explored.

### SST, in situ temperature, salinity, and dynamic height

To understand the driving mechanism for the TIW-modulated Chl-a change and the linkage between TIW-modulated Chl-a and the TIW-related temperature and salinity changes in the Equatorial Pacific Ocean, we also examined and analyzed the SST and in situ temperature (T) and salinity (S) data, as well as in situ dynamic height data.

The daily NOAA high-resolution blended SST data^38^ are gap-free products from using the optimum interpolation (OI) technique. The blended SST data are produced from various sources such as sea ice datasets, in situ data from ships and buoys, and the Pathfinder Advanced Very High Resolution Radiometer (AVHRR) SST data. The spatial resolution for this product is of 1/4°.

The TAO is a set of moored buoy arrays across the Equatorial Pacific Ocean that help to better understand and predict climate variations related to the El Niño and Southern Oscillation (ENSO) (https://www.pmel.noaa.gov/gtmba/pmel-theme/pacific-ocean-tao). The TAO project has been a multi-national effort to provide data in real-time for climate research and forecasting in the upper 500 m of ocean since the 1980s. The measurements include temperature, salinity, wind speed and direction, current, long wave radiation, etc.

In this study, the in situ temperature, salinity, and dynamic height of the TAO measurements, which are obtained at the location [140°E, 0°N] in 2020, are used to study the linkage between the temperature and salinity variability driven by the TIW and the TIW-driven Chl-a variability, and further explore the mechanism for the TIW-modulated Chl-a change in the Equatorial Pacific Ocean. It is noted that dynamic height is a measure of the elevation of the sea surface (without the ocean waves) calculated by integrating the specific volume anomaly of the sea water between the surface and the depth at 500 m.

## Results

### Maps of SST and Chl-a in the Equatorial Pacific Ocean

**Figure 1:**
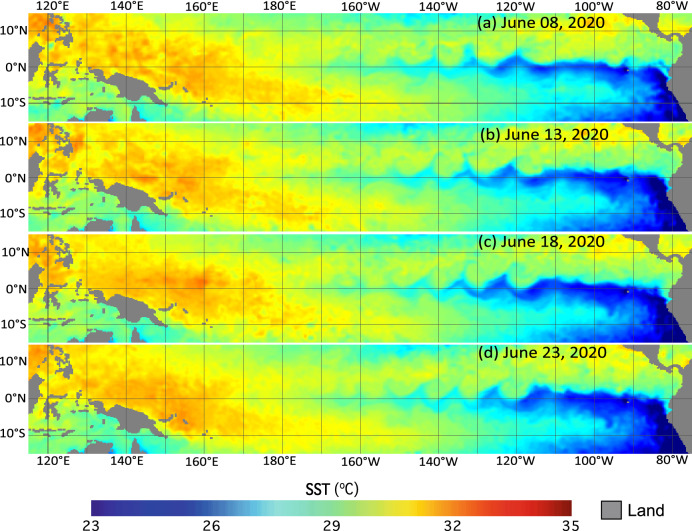
Satellite-measured SST in the Equatorial Pacific Ocean on (**a**) June 8, 2020, (**b**) June 13, 2020, (**c**) June 18, 2020, and (**d**) June 23, 2020.

Figure 1 shows SST maps in the period from June 8–23, 2020, with a gap of five days. Indeed, the Equatorial Pacific Ocean exhibited the cusp-shaped cold SST anomaly patterns driven by the TIW^1,4^. The SST pattern modulated by the TIW extended from 80 to 160ºW in the eastern and central Equatorial Pacific Ocean. The cusp-shaped cold SST anomaly was about 2–3°C lower than that in the neighboring region, and the wave length of the wave-shaped SST pattern could reach ~ 10 longitudinal degrees wide.

Figure 1a–d shows that the cold SST anomaly pattern propagated westward. As an example, the cold SST anomaly, which was located around 120ºW, moved westward on June 13, 2020 (Fig. 1b) and June 18, 2020 (Fig. 1c). On June 23, 2020, this cold SST anomaly pattern was located at 125ºW. In comparison to the SST pattern in the eastern Pacific Ocean, SST in the western Pacific Ocean was less affected by the TIW with no wave-shaped cold SST anomaly patterns.

**Figure 2:**
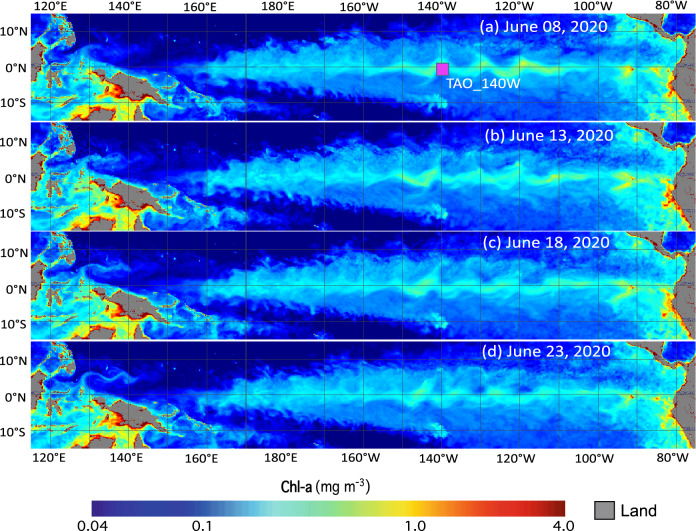
VIIRS-measured Chl-a in the Equatorial Pacific Ocean on (**a**) June 8, 2020, (**b**) June 13, 2020, (**c**) June 18, 2020, and (d) June 23, 2020. Note that the solid pink square in panel 2a marks the location of the TAO_140W mooring at [140°W, 0°N] for further data analysis.

Similar to SST, VIIRS Chl-a maps from June 8–23, 2020, are also examined (Fig. 2). The maps show that the Chl-a spatial pattern in the Equatorial Pacific Ocean was also modulated by the TIW. The cusp-shaped enhanced Chl-a extended from eastern to central Equatorial Pacific Ocean. The enhanced Chl-a reached ~ 0.3 mg/m^3^, while Chl-a in the neighboring ocean region were only ~ 0.1 mg/m^3^.

**Figure 3:**
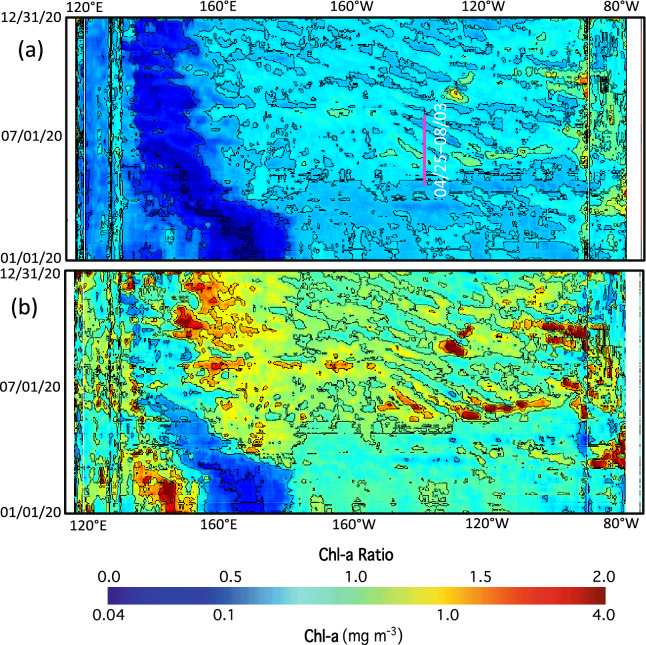
(**a**) Temporal change of Chl-a along the equator between 115°E and 75°W in 2020 and (**b**) temporal change of normalized Chl-a ratio (Chl-a/Chl-a^(*Mean*)^) along the equator between 115°E and 75°W in 2020. The pink line in the panel 3a marks the period of April 25–August 3, 2020, at the TAO_140W station for further data analysis.

Figure 2a–d shows that the Chl-a pattern was not static. Instead, it propagated westward. On June 8, 2020, enhanced Chl-a over ~ 0.3 mg/m^3^ could be found around 140ºW in the Equatorial Pacific Ocean (Fig. 2a). Five days later on June 13, 2020, the high Chl-a were located to the west of its original location (Fig. 2b). It further propagated westward on June 18, 2020 (Fig. 3b). On June 23, 2020, it was already at 145°W. Thus, the cusp-shaped high Chl-a pattern propagated westward about 5 longitude degrees in 15 days from June 8, 2020, to June 23, 2020.

Figures 1 and 2 show that both SST and Chl-a are modulated by the TIW in the Equatorial Pacific Ocean. The cooler SST and enhanced Chl-a were reasonably well matched to each other in terms of the spatial extent and pattern, propagation speed, etc. As an example, a cold SST anomaly (Fig. 1a) and high Chl-a (Fig. 2a) were both located at 120ºW on June 8, 2020, along the equator even though the distributions of the cold SST anomaly and high Chl-a were not exactly the same. From June 8–23, 2020, the cold SST anomaly pattern was found propagating westward with little change in terms of its pattern. For Chl-a, the high Chl-a pattern was distorted in this period even though the westward propagation of high Chl-a could still be found in Fig. 2. On the other hand, the cold SST anomaly pattern was insignificant to the west of 180°E (Fig. 1). In comparison, the distorted Chl-a further extended to 160°E as shown in Fig. 2.

### TIW-modulated Chl-a in 2020

To further characterize and quantify Chl-a dynamics driven by the TIW, Chl-a changes along the equator between 115°E and 85°W in 2020 are analyzed. In 2020, the Multivariate ENSO Index (MEI) was ~ –1.0 to 0 (https://psl.noaa.gov/enso/mei/). As a reference, MEI reached over +2.0 in the El Niño event in 2015 and it was below –2.0 in the La Niña event in 2011. This implies that the TIW strength in 2020 was slightly higher than that in a normal year. The time-longitude plot of Chl-a (Fig. 3a) shows that Chl-a along the equator were modulated by the TIW between May 2020 and December 2020. Indeed, the enhanced Chl-a feature can be found between 90°W and 160°E. The high or low Chl-a driven by the TIW propagated westward. Specifically, high and low Chl-a could be over ~ 0.5 mg/m^3^ and ~ 0.1 mg/m^3^, respectively. In a period of half a year, a low and high Chl-a pattern propagated from 110°W to 180°E.

In order to further quantify Chl-a dynamics modulated by the TIW, the mean Chl-a (denoted as Chl-a^(*Mean*)^) during the entire 2020 year at each pixel along the equator are calculated, and ratios of Chl-a/Chl-a^(*Mean*)^ as the normalized Chl-a variability along the equator in 2020 are shown in Fig. 3b. Similar to the propagation of Chl-a patterns, the westward propagation of Chl-a/Chl-a^(*Mean*)^ ratio can easily be identified in Fig. 3b. The ratio of Chl-a/Chl-a^(*Mean*)^ modulated by the TIW normally ranged from about 0.7 to 1.3 at locations between 100°W and 180°W. At some locations, such as around 130°W, the ratio of Chl-a/Chl-a^(*Mean*)^ even reached ~ 2.0 in early September 2020. This shows that the TIW can cause about ±30% of Chl-a variability in terms of the Chl-a variance relative to its corresponding mean Chl-a value in the eastern and central Equatorial Pacific Ocean.

**Figure 4:**
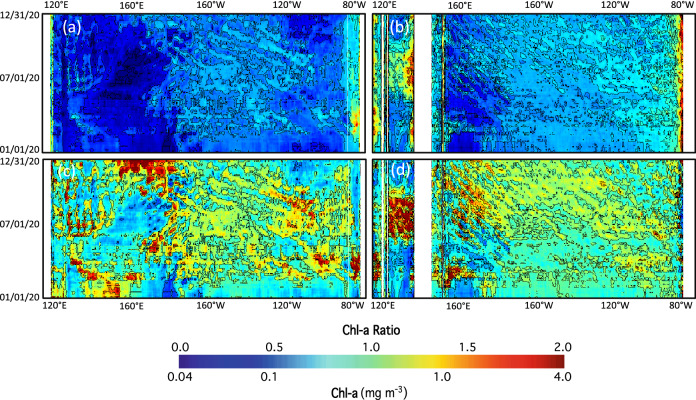
(**a**) Temporal change of Chl-a at 5°N between 115°E and 75°W in 2020, (**b**) temporal change of Chl-a at 5°S between 115°E and 75°W in 2020, (c) temporal change of Chl-a ratio (Chl-a/Chl-a^(*Mean*)^) at 5°N between 115°E and 75°W in 2020, and (d) Chl-a ratio at 5°S between 115°E and 75°W in 2020.

In Fig. 2, Chl-a modulated by the TIW occurred not only along the equator, but also beyond the equator. At 5°N, the modulation of Chl-a was still observed (Fig. 4a). The westward propagation of the TIW-driven Chl-a distortion occurred between 110°W and 160°W. At 5°N, Chl-a generally were less than those along the equator ranging from ~ 0.1–0.2 mg/m^3^. In fact, the extent of Chl-a modulated by the TIW at 5°N was less than that along the equator. Figure 4c shows the normalized Chl-a variability (Chl-a/Chl-a^(*Mean*)^) at 5°N. The ratios were in the range of 0.7–1.3 in 2020. Thus, the modulation of Chl-a at 5°N is similar to that along the equator even though the magnitude of Chl-a variability was smaller at this location.

At 5°S, Chl-a modulated by the TIW extended from 120°W and 160°E (Fig. 4b). Similar to Chl-a at 5°N, Chl-a ranged from ~ 0.1–0.2 mg/m^3^ across 5°S. The Chl-a distortion driven by the TIW was ~  ±25–30% of the mean Chl-a value. Propagation of the TIW-driven Chl-a variability was also notable at this latitude in Fig. 4b and d. However, the propagation speed across 5°S was slower than that across 5°N and the equator. As an example, the TIW-driven high/low Chl-a traversed the eastern Pacific Ocean from 120°W to 160°W in 6 months at 5°S, while it only took ~ 3–4 months for the TIW-driven high/low Chl-a to cross the same distance at 5°N. It is also noted that the Chl-a distortion at 5°S between 180°E and 160°E was initiated at the central Equatorial Pacific Ocean. In comparison, the TIW that modulated Chl-a at 5°N and the equator started in the eastern Equatorial Pacific Ocean.

### Mechanism for the TIW-modulated Chl-a in 2020

To investigate the mechanism that drives the Chl-a change, the TAO measurements at 140°W along the equator are examined to identify the linkage of the changes in physical parameters driven by the TIW and the corresponding Chl-a changes. As shown in Fig. 3a, Chl-a in the period between late April and mid-May 2020 were not affected by the TIW. Starting in late May 2020, Chl-a at this location began to be modulated by the TIW, and the distorted Chl-a pattern propagated westward.

**Figure 5:**
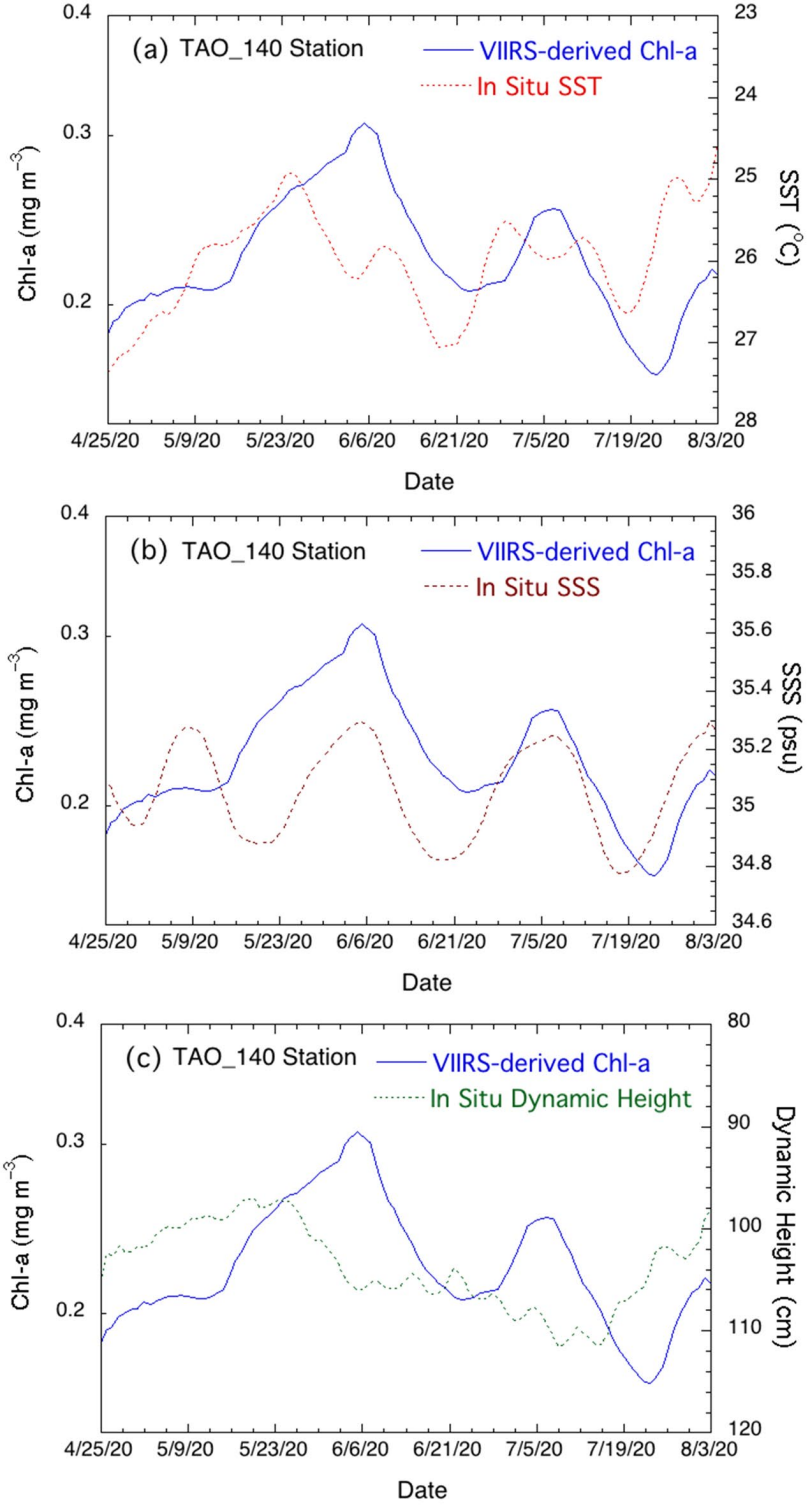
Temporal change of VIIRS-measured Chl-a in comparison with (**a**) in situ SST variation, (**b**) in situ SSS variation, and (**c**) dynamic height from April 25–August 3, 2020 at the TAO_140W station. Note the Chl-a, SST, SSS, and dynamic height are 5-day running mean values in order to remove the measurement noises of these parameters. The y-axis values (right side) for plots SST (**5a**) and dynamic height (**5c**) are scaled from high to low to show better comparisons of their variations with those of Chl-a (scaled left).

Figure 5a shows the changes of Chl-a and SST in the period of April 25–August 3, 2020 (time range marked in Fig. 3a). In early June, Chl-a reached over ~ 0.3 mg/m^3^, while SST was at ~ 26°C. In the period between June 6–21, Chl-a dropped to ~ 0.2 mg/m^3^. In comparison, SST reached its lowest in late May and gradually increased to over 27°C on June 17. In the period between June 2–28, SST dropped from over 27 to 25.5°C and stayed at ~ 25.5‒26°C for several days. Meanwhile, Chl-a at this location increased from ~ 0.2 mg/m^3^ to 0.25 mg/m^3^ from June 24–July 5. In the period between July 5–22, Chl-a dropped from 0.25 to 0.17 mg/m^3^, while SST increased from < 26°C to 26.6°C from late June to mid-July. However, in the period between July 19–August 3, SST dropped from 26.6 to 24.5°C. Correspondingly, in the same period Chl-a increased from about 0.17 to 0.20 mg/m^3^.

Figure 5a shows that the Chl-a change at this location was more or less inversely related to the change of SST even though the increase/decrease of Chl-a was not exactly in phase with the SST decrease/increase at this location. In general, the Chl-a change lags the SST change for a couple of days. The enhanced Chl-a modulated by the TIW matched reasonably well with the cooler SST and vice versa. Indeed, the in situ observations of SST and Chl-a in this period are consistent with the observations in Figs. 1 and 2.

Figure 5b shows SSS changes in the same time period in comparison to Chl-a changes. Before late May, there was no linkage between the SSS variation and Chl-a change. However, SSS and Chl-a changes were highly related with the start of the TIW since late May. In the period between late May and early August 2020, three Chl-a peaks on June 6, July 5, and August 3 were coincident with SSS peaks. On the other hand, the two troughs of Chl-a both occurred in the period of low SSS values with ~ 2–3 days lag.

Figure 5c shows comparisons of the change in Chl-a and the change in the dynamic height. Obviously, peaks/troughs of Chl-a and the troughs/peaks of the dynamic height are not exactly in phase, and there is an apparent phase difference between the two for ~ 7–9 days. This is similar to the changes of Chl-a and SST as shown in Fig. 5a. As an example, Chl-a trended lower from mid-June after the dynamic height increased from late May to early June. The dynamic height reached its maximum in mid-July, while the Chl-a minimum occurred on July 24.

In addition, the relationships between the Chl-a change and changes of SST, SSS, and dynamic height were also quantitatively examined. The cross-correlation coefficients between Chl-a and SST, Chl-a and SSS, and Chl-a and dynamic height are –0.46 (anti-correlation), 0.74, and –0.58 (anti-correlation), respectively, with the corresponding time lag of about 7 days, 1 day, and 8 days, respectively. These cross-correlation coefficients and quantitative results in Fig. 5 demonstrate that Chl-a change is related at least to the TIW-driven changes of SST, SSS, and dynamic height. Furthermore, the biological activity generally lags the TIW-driven physical variability. This also provides solid evidence why the enhanced Chl-a patterns in Fig. 2 are different from the corresponding cooler SST patterns in Fig. 1 even though both are associated with the TIW.

**Figure 6:**
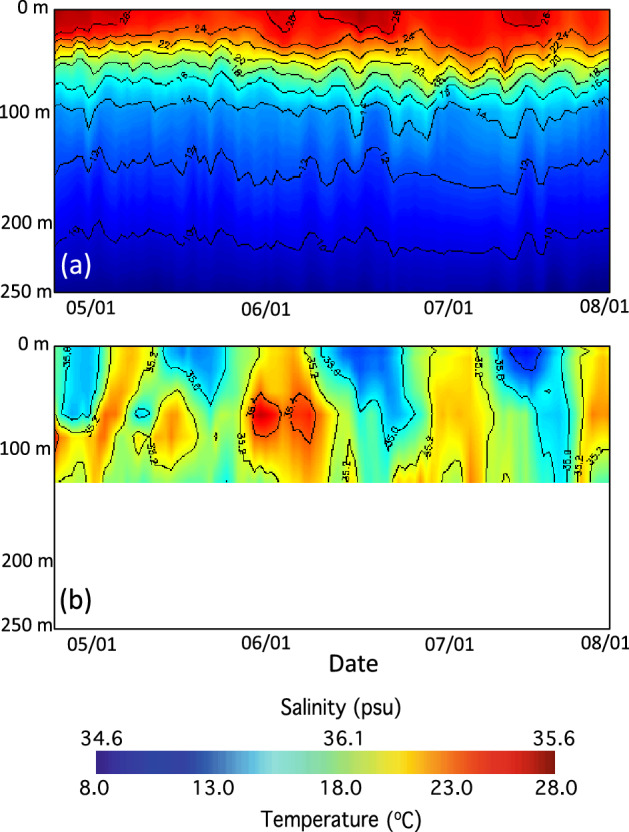
Profiles in the upper 250 m at the TAO_140W station from April 25–August 3, 2020 for (**a**) temperature and (**b**) salinity. Note that there were no measurements of salinity below 120 m depth at the TAO_140W station.

Figure 6 shows the vertical distributions of water temperature (Fig. 6a) and salinity (Fig. 6b) in the period between April 24–August 3, 2020. In this period, SST in Fig. 5a represented the temperature changes in the upper 20 m layer. On the other hand, the 24°C isotherm depth changes were found to be related to the Chl-a variation. Chl-a increased following the deepening of the 24°C isotherm depth, while Chl-a decreased following the shoaling of the 24°C isotherm depth. In the period before late May, the 24°C isotherm was relatively flat at ~ 25 m. From late May to early June, the 24°C isotherm deepened. In fact, this happened with the increase of Chl-a. From early to late June, the 24°C isotherm shoaled up to 10 m. In this period, Chl-a dropped from over ~ 0.3 to ~ 0.2 mg/m^3^. From late June to early July, the 24°C isotherm deepened again. In the meantime, Chl-a showed an increase to ~ 0.25 mg/m^3^ correspondingly in this time frame. In early July, the 24°C isotherm became stabilized and then slowly shoaled up until late July. Similarly, Chl-a dropped from ~ 0.25 to ~ 0.17 mg/m^3^. In late July, Chl-a at this location increased with the deepening of the 24°C isotherm.

The vertical distribution of salinity (Fig. 6b) shows that the periodical change of the salinity can reach to ~ 50 m deep. This is the depth of the 20–24°C isotherm depth as shown in Fig. 6a. The salinity profile at this location shows that salinity increased with the depth. As an example, salinity was ~ 34.8 psu at the surface, and it increased to 35.2 psu in mid-May. In May 2020, the 24°C isotherm depth was generally flat, showing that the salinity variation in the period was not related to the TIW-driven 24°C isotherm depth change. Starting in late May 2020, the fluctuation of the surface-layer salinity at this location generally followed the change of the 24°C isotherm depth, i.e., salinity increased/decreased with the deepening/shoaling of the 24°C isotherm. It is also noted that the TIW-driven Chl-a change in 2019 (results not shown) in the eastern Equatorial Pacific Ocean was also in phase with the changes of salinity and the 24°C isotherm depth as shown in 2020.

The TIW is not only featured with the anomalous SST and SSS patterns at the sea surface, but also causes the subsurface variability of the temperature and salinity^12^. Figure 6a shows that the TIW was also manifested with the subsurface temperature changes between late May and August 3, 2020. The meridional current measurements at this station in the subsurface layer was also found to contribute to the salinity changes (results not shown). The positive (northward) meridional current coincided with the increasing salinity and deepening the 24°C isotherm. The negative (southward) meridional current coincided with the decreasing salinity and shoaling of the 24°C isotherm. In the TIWs, the water horizontal and vertical movement and subsurface mixing indeed led to the temperature and salinity changes at the surface layer and subsurface layer. Consequently, the Chl-a change is driven by various ocean processes such as meridional current variability, vertical water movement, and isotherm depth variations, i.e., upwelling and downwelling, associated with the TIWs.

Nutrients such as nitrate and phosphate are critical for the algal growth and the phytoplankton bloom. However, there were no nutrient measurements at the TAO buoy moorings. In the eastern Equatorial Pacific Ocean, nutrient concentrations increase with the depth in this region^39^. As an example, the nitrate concentration increases from ~ 4‒6 μmol/kg at the surface to ~ 5‒15 μmol/kg at the depth of 50 m in the eastern Equatorial Pacific Ocean. On the other hand, the relationship of the change in salinity and the variability of the meridional current suggests that water of increasing salinity brought by the positive (northward) meridional current is also more nutritious, and consequently, enhances the phytoplankton growth. Thus, the physical processes, e.g., water movement and subsurface mixing, not only can cause water temperature and salinity changes, but also lead to the changes of the sea surface nutrient level in the Equatorial Pacific Ocean.

It is noted that there are phase shifts between the changes of Chl-a and SST, and between the changes of Chl-a and dynamic height, as shown in Fig. 5a and c, while the change of Chl-a is in phase with that of SSS. Similar to the nutrient property, salinity is a conservative ocean property which is generally not affected by various ocean and atmospheric processes such as solar radiation, air-sea interaction, etc. Consequently, SSS and nutrients are highly correlated. Therefore, SSS can serve as a surrogate for the nutrient levels in the region, i.e., high/low salinity is associated with enhanced/dampened nutrient concentrations. This is consistent with the reports that the TIWs drive elevated nutrient concentration along the equator^40,41^. Thus, phytoplankton growth is in phase with the SSS as shown in Fig. 5b.

The iron concentration has been found to be less or even negatively affected by the TIWs^41,42^. Thus, it could not contribute to the enhanced Chl-a as observed in this study. In comparison to SSS and nutrient concentrations, water temperature and the 24° isotherm depth are impacted by various ocean and atmospheric processes which were mentioned above and generally do not have impact on the salinity and nutrients. This suggests that SST and dynamic height are not conservative ocean properties, and they are only moderately related to the nutrient concentration and Chl-a. The ocean and atmospheric processes may also lead to the time lags for the changes in Chl-a versus SST and Chl-a versus the 24° isotherm depth.

In essence, the salinity time series at the TAO_140 station in Figs. 5b and 6b generally represents the nutrient changes in the period from April 24–August 3, 2020. The Chl-a variation is consistent with the supply of the nutrients which were actually modulated by the TIW-driven variability in the subsurface layer. The subsurface horizontal and vertical water movement and mixing within the deepening 24°C isotherm depth increased nutrients and led to the enhanced Chl-a. On the other hand, the sea surface nutrient level was low when the 24°C isotherm depth shoaled up, and consequently Chl-a decreased due to the depressed nutrient concentrations.

## Conclusion

The previous studies on the physical aspect of the TIW such as SST, SSS, and velocities have been well explored. Even though the impact of the TIWs on Chl-a was reported, it was difficult to be thoroughly examined and quantified due to data gaps in satellite Chl-a products caused by frequent cloud coverage and other unfavorable conditions^34,35^. In particular, the mechanism that drives the biological activity with the TIW is still not well understood. In this study, VIIRS-derived daily gap-free Chl-a data taken in 2020 are used to characterize and quantify the biological variability modulated by the TIW in the eastern and central Equatorial Pacific Ocean. Chl-a in the central and eastern Equatorial Pacific Ocean were significantly affected by the westward propagation of the TIW from late May to the end of 2020. The Chl-a change driven by the TIW covers in the region between around 5°S and 5°N and stretches from 90°W to 160°E. The disturbance of Chl-a driven by the TIWs accounted for about ±30% of the corresponding Chl-a values in each location.

This study shows that the spatial pattern of the enhanced Chl-a matched reasonably well with the cooler SST in the Equatorial Pacific Ocean driven by the TIW. The westward propagating speed of the enhanced Chl-a is consistent with the westward propagating speed of the TIW-driven SST. In order to study the driving mechanism for the TIW-driven Chl-a variability, the TAO in situ measurements of water temperature, salinity, and dynamic height at 140°W along the equator are investigated and compared with the corresponding VIIRS Chl-a time series at the same location. After the start of the TIW in late May, the Chl-a change was generally in phase with the change of SSS. The cross-correlation coefficients with the highest magnitude between Chl-a and SST, Chl-a and SSS, and Chl-a and dynamite height are –0.46, + 0.74, and –0.58, respectively, with the corresponding time lag of 7 days, 1 day, and 8 days, respectively. In fact, the peak Chl-a matched well with the peak of SSS. Examination of water temperature and salinity profiles at the TAO station shows that the change of SSS is linked to the perturbation of the 24°C isotherm depth due to the water movement and mixing process driven by the TIW.

In the TAO mooring stations, nutrient concentrations are not measured. Using the salinity as a surrogate for the nutrient level in the region, we show that the Chl-a variability as observed by VIIRS was caused by the changes of the nutrient level due to the TIW. When the 24°C isotherm depth deepens, vertical mixing in the subsurface brought higher nutrient levels and salinity water to the surface layer, and consequently boosted the phytoplankton growth and led to the enhanced Chl-a. On the other hand, the nutrient level as well as salinity in the surface layer decreased and Chl-a dampened when the 24°C isotherm depth shoaled up. Indeed, the in situ measurements at the TAO not only can be used to study the physical processes in the Equatorial Pacific Ocean, but also provides valuable insight on the mechanism that the TIWs modulate ocean biological activities in the region.
